# Supercritical Fluid Extraction of Celery and Parsley Fruit-Chemical Composition and Antibacterial Activity

**DOI:** 10.3390/molecules25143163

**Published:** 2020-07-10

**Authors:** Dusan Misic, Vanja Tadic, Malgorzata Korzeniowska, Jakov Nisavic, Ksenija Aksentijevic, Jelena Kuzmanovic, Irena Zizovic

**Affiliations:** 1 Faculty of Biotechnology and Food Science, Wroclaw University of Environmental and Life Sciences, 51-651 Wroclaw, Poland; malgorzata.korzeniowska@upwr.edu.pl; 2 Faculty of Veterinary Medicine, University of Belgrade, 11000 Belgrade, Serbia; jakovmoni@vet.bg.ac.rs (J.N.); pojavica@gmail.com (K.A.); 3 Institute for Medicinal Plant Research ‘‘Dr. Josif Pancic’’, 11000 Belgrade, Serbia; vtadic@mocbilja.rs; 4 Center for Food Analyses, Department of Microbiology, 11000 Belgrade, Serbia; jelena.z.kuzmanovic@gmail.com; 5 Faculty of Chemistry, Wroclaw University of Science and Technology, 50-373 Wroclaw, Poland; irena.zizovic@pwr.edu.pl

**Keywords:** celery, parsley, supercritical extraction, antibacterial activity, cytotoxicity

## Abstract

Supercritical fluid extraction as an environmentally friendly technology was applied to isolate biologically active extracts from celery and parsley fruits for potential applications in the food industry. The extractions were performed under mild temperature conditions of 39.85 °C and at pressures of 10 and 30 MPa. The extracts were analyzed regarding their chemical composition, antibacterial activity, and cytotoxic effect. Sedanolide was the dominant component of the celery fruit extracts, comprising more than 70% of the obtained fraction, while the content of apiole in the parsley fruit SC CO_2_ extracts exceeded 85%. The celery fruit extracts showed strong and moderately strong antibacterial activity against tested *Staphylococcus aureus*, *Bacillus (B.) cereus*, *B. subtilis*, *B. circulans*, *Listeria* (*L*.) *greyi*, *L. seeligeri* and *L. welshimeri*, with minimal inhibitory concentration (MIC) values between 160 and 640 µg/mL, and weak activity against the selected *Salmonella* isolates with a MIC of 2560 µg/mL. The parsley extract obtained at 10 MPa showed strong and moderately strong antibacterial effects against *Bacillus* strains with obtained MICs of 160–640 µg/mL, and weak activity against *Staphylococcus, Listeria*, and *Salmonella* with a MIC of 2560 µg/mL. Cytotoxicity investigation showed that the extracts with proven antibacterial activity had no cytotoxic effect on rabbit kidney cells at concentrations of up to 640 µg/mL.

## 1. Introduction

Humanity is in desperate need of novel antibacterial molecules. Due to the significant consequences of antibiotic resistance over the past years, studies of the antimicrobial activity of natural bioactive molecules have become deeply important. With no new broad-spectrum antibiotic classes having appeared on the market for over 30 years, bioactive molecules from natural sources remain the sole means of producing new compounds. Nowadays, food quality and safety standards are stricter than in the past. Many states have implemented serious revisions of existing laws on food production and on the reproduction of plants and animals. Substances allowed in food production for decades are now banned and/or being phased out [1,2]. Natural bioactive compounds are now getting more space in the food industry to prevent the propagation of food-contaminating bacteria or to prevent the spread of foodborne diseases [1,3].

Supercritical extraction is a superior “green chemistry” technology which is used for the isolation of herbal extracts without the need for organic solvents [4,5]. The most striking advantage is its low operating temperatures, making it possible to preserve thermo-degradable compounds and easily separate extracts from supercritical carbon dioxide (SC CO_2_) by pressure reduction. The superiority of this technology is also reflected in the possibility of modeling the conditions for the production of standardized extracts with desirable attributes; by changing the extraction parameters, unwanted molecules may be eliminated, and concentrations of desired molecules may be enhanced. The preservation of active components due to the mild processing conditions and the solvent-free nature of the supercritical extracts makes this approach particularly suitable for application in the food industry.

It has been shown that some SFE herbal extracts have extraordinary antibacterial activity, but the organoleptic properties or toxicity of these extracts has limited their wider use. Typical examples are *Usnea barbata* (*L.*) *Weber ex F. H. Wigg.* (Parmeliaceae) supercritical extract, which has not been approved by FDA because of its hepatotoxicity, and hop (*Humulus lupulus* L., Cannabaceae) supercritical extract, whose usage is limited due to its strong bitterness; both have shown extremely strong antibacterial activity against Gram-positive bacteria [6,7].

The advantages of celery and parsley SFE could therefore be due to their specific taste and aroma, traditionally used every day all over the world in all branches of the food industry (e.g., juices, teas, soups and meat products).

Celery (*Apium graveolens* L.) and parsley (*Petroselinum crispum* (Mill.) Fuss), members of the Apiaceae family, are commonly known and used as culinary herbs, mostly due to their flavoring properties that originate mainly from the essential oils in the plant. One of the main compounds responsible for the characteristic aroma of celery is sedanolide [8,9], while two others, sedanenolide and 3-n-butylphthalide, both phthalides, can also be present in high concentrations [10,11,12,13]. Parsley seed essential oil is composed mainly of allylpolyalkoxybenzenes, myristicin, apiole, and 2,3,4,5-tetramethoxyallylbenzene, which give parsley its distinct odor and flavor [4,14].

Despite their frequent use in the food industry, very few data on SFE from celery and parsley fruits can be found in the scientific literature. A comparative analysis of the chemical composition of celery fruit essential oil showed that SFE was superior to hydrodistillation regarding both yields and concentrations of the main active compounds, e.g., sedanenolide, sedanolide and 3-*n*-butylphthalide [11,13,15,16,17]. Investigations on the antibacterial activity of the SFE from celery seeds collected at 10 MPa have already been published, and activity against *Staphylococcus*, *Listeria* and *Helicobacter pylori* was reported [16,18]. But to the best of our knowledge, there is no data in the literature on the SFE from celery fruit at 30 MPa. Also, there are either no reports available on the chemical and biological properties of parsley seed extracts obtained by SFE, or on the cytotoxic effects of celery and parsley fruit oils.

The aim of this work was to analyze the antibacterial activity and cytotoxic effect of celery and parsley fruit extracts obtained by SFE in order to explore the possibility their use not only as flavoring, but also as natural antibacterial agents. Extractions were performed at a mild temperature of 313 K (39.85 °C) at two pressure levels. At the lower pressure, i.e., 10 MPa, mostly the essential oil contained in the fruit secretory ducts was expected to be extracted, while at the higher pressure, i.e., 30 MPa, coextraction of the fatty oils in the seeds may take place as well. To the best of our knowledge, there are no published data on the properties of celery and parsley fruit extracts obtained by SFE at pressures higher than 20 MPa.

## 2. Results

The processing yields obtained by SFE from celery and parsley fruits are presented in Table 1. The results revealed that raising the pressure range did not influence the yield much from celery. In the case of parsley, a substantially higher yield was obtained at 30 MPa than at 10 MPa. The exhaustion of the plant material in the applied experimental setup was determined using the mass ratio of CO_2_ consumed and plant material placed in the extractor, i.e., 24 for celery and around 20 for parsley for the both SFE conditions.

The results of the GC/MS analyses of the SC CO_2_ extracts are presented in Table 2 and Table 3. No significant differences in the chemical composition of investigated fractions obtained at different pressures were found. In the celery extracts, the dominant component was sedanolide, i.e., 72.24 and 73.69% at 10 and 30 MPa, respectively. Almost identical amounts of 3-butyl phthalide, β-selinene, α-selinene, (*E*)-caryophyllene and pinocarvyl acetate were detected in both extracts (Table 2). However, limonene was present in a higher quantity in CE1 (celery extract obtained at 10 MPa) than in CE2 (celery extract obtained at 30 MPa), i.e., 7.38 and 4.14%, respectively. In the case of the parsley extracts, apiole was the main component, i.e., 86.06% at 10 MPa, and 88.90% at 30 MPa. Camphene and β-pinene, although identified in PE2 (obtained at 30 MPa), were not detected in the parsley extract obtained at 10 MPa (PE1). Elemicin, 2,3,4,5-tetrametoxyallyl benzene, carotol, sedanenolide and murolan-3,9(11)-diene-10-peroxy were present only in PE1. Among these constituents, only sedanenolide was detected in significant amounts (2.06%).

The results of the antibacterial activity investigation are presented in Table 4. Both celery extracts showed antibacterial activity against all tested strains with obtained MIC values 160 µg/mL and 320–640 µg/mL (Table 4). The antibacterial activities of both celery extracts (CE1 and CE2) were almost unified against *Bacillus cereus* strains with MICs of 160–320 µg/mL. Extract CE1 had a slightly stronger effect on staphylococci with MICs of 160–320 µg/mL than the extract CE2, with MICs of 320–640 µg/mL. Almost identical results regarding the antibacterial activity of CE1 and CE2 were obtained against *Listeria* species, with MICs of 640 µg/mL, except in the case of *L. welshimeri*, where the MIC of CE1 was halved (320 µg/mL). Celery extracts also showed activity against *Salmonella* with a MIC of 2560 µg/mL. Parsley extract collected at 10 MPa (PE1) showed antibacterial activity against investigated *Bacillus* spp., with MICs of 160–640 µg/mL. The activity of PE1 was significantly weaker against staphylococci and *Listeria* compared to CE1, with the obtained MIC of 2560 µg/mL, whereby the same MIC of PE1 was obtained against *Salmonella*. The parsley extract collected at 30 MPa (PE2) showed activity against the tested bacilli with an obtained MIC of 2560 µg/mL, except the one against *B. circulans*, with a MIC of 320 µg/mL. Extract PE2 showed no activity in the investigated concentrations against any of the remaining tested strains. For all *Bacillus* isolates, sedanolide MIC values were 1280 µg/mL and limonene MIC values were 2560 µg/mL. The obtained MIC values indicated that sedanolide had a weaker effect than CE1, CE2 and PE1 and a stronger effect than PE2 on *Bacillus* spp. Limonene had a weaker effect than CE1, CE2, PE1 and an identical effect to PE2 on *Bacillus* isolates. The obtained MIC values of sedanolide against *S. aureus* were 2560 µg/mL, i.e., weaker than CE1 and CE2. The obtained MIC values of limonene against *S. aureus* were >2560 µg/mL, i.e., at the given concentration, limonene had no effect on staphylococci. Against other investigated isolates, sedanolide and limonene showed no activity at the given concentrations.

The obtained results of cytotoxic activity for CE1 and PE1 are presented in Table 5. CE1 and PE1 at a concentration of ≤320 µg/mL had no toxic effect on the exposed cells. At a concentration of 640 µg/mL, weak cytotoxicity was shown, i.e., with approximately 12% cell death (survival rate ≥88%). At maximum concentrations of 2560 µg/mL, more than 50% of the exposed cells still survived exposure to both extracts.

## 3. Discussion

Essential oils are typically complex mixtures, having a few to several hundred components, classified mainly as hydrocarbons, oxygenated compounds (monoterpenes and sesquiterpenes), phenylpropanoids, diterpenes, etc., which define their specific odor and flavor. Beside their aromatic properties, essential oils possess significant antimicrobial properties, which enable their application in the pharmaceutical, food, cosmetic, and fragrance industries. The chemical profiles of essential oils are dependent on the extraction method used, as well as on other factors. The investigated essential oils revealed different qualitative and quantitative compositions when the most widely used technique for extraction, hydrodistillation, was applied. Limonene was the major constituent in the celery seeds essential oil [13], while in our samples of CE1 and CE2, phthalide derivatives were the most abundant compounds (sedanolide, representing 72.24 and 73.69% of CE1 and CE2, respectively). It is worth mentioning that phthalide constituents are known as potent biologically active substances [13,20]. A chemical profile analysis of parsley seed essential oils revealed differences, mainly in apiole and myristicin contents (Table 3). Namely, apiole represented the major component, with percentages greater than 80% in our samples (86.08 and 88.90% in PE1 and PE2, respectively), while the essential oils obtained by hydrodistillation contained myristicin in significantly greater quantity in comparison to the investigated PE1 and PE2 [21] essential oils (the apiole content was less than 60%). Saqqa and co-authors [22] found that the essential oil obtained by hydrodistillation from parsley seeds contained mainly myristicin, and apiol was present at a quantity of less than 20%. The significant difference in the yield of SFE from parsley extracted at 10 and 30 MPa obtained in this study may be due to the higher solubility of parsley secondary metabolites in SC CO_2_ at 30 MPa, as well as, by the coextraction of triglycerides from the seed part of the fruit under the higher pressure.

The modern food industry still faces the problem of how to produce foods that are free from foodborne pathogens and food spoilage agents, that is, how to provide a prolonged expiration date of the product. Some foodborne pathogens are normally present in animals, or may remain “invisible” due to asymptomatic infection, meaning that the risk of their retention during food production is high (*Campylobacter* in pigs and poultry, *Salmonella* in poultry, *E. coli* O157:H7 in cattle, enterotoxin producing *S. aureus* in almost all animals) [1]. All foodborne pathogens from animals can be found on plants that have been fertilized with manure of animal origin. The treatment of livestock with antibiotics in order to control the presence of pathogenic bacteria before their entry into the food chain production is prohibited [1]. All legally established regulations require microbiologically safe food, and this means the complete absence of foodborne pathogens. In contrast, in some types of food (i.e., meats), the presence of a certain number of nonpathogenic and opportunistic bacteria (including some members of the Enterobacterales family) is inevitable, and thus, is allowed. These bacteria continuously multiply and shorten the shelf life of food. Supercritical extracts might considerably improve technological processes of food production; there are a few reasons for this. Namely, supercritical extracts are entirely natural products; this point is extremely important for applicable legislation, market demand and consumer requirements. Additionally, in some cases, supercritical extracts of vegetables and fruits might be used directly in the production process, avoiding the high probability of microbiologically contaminated raw crops. An example is beer production, where supercritical hops extracts have completely or partially replaced hops cones [7]. In addition, many extracts obtained by SFE have antibacterial activity that can further effectively prevent the multiplication of pathogenic and food spoilage bacteria, thus extending the shelf life but also ensuring food safety.

In this study, the antibacterial activity was analyzed of celery and parsley (seeds) supercritical extracts (SFE) against bacteria that cause food spoilage or alimentary infections and intoxication. Particularly important in this study are sporulating bacteria, especially *B. cereus*, whose spores can survive processing, including heat treatment, and subsequently, may germinate and turn to vegetative forms, which produce toxins. There is still no consensus on what is considered a strong, moderate or weak antimicrobial effect of a herbal extract, and thus, interpretations are mostly the result of the arbitrary views of the authors (concentrations of extracts expressed in µg/mL, mg/mL, mg/L, g/L, µM, mM, M and ppm) [23]. In several papers, however, the authors have noted that the potencies of plant extracts should not be compared with those of antibiotics due to their completely different chemical natures, exhibiting mechanisms of action according to the presence of secondary metabolites; rather, the potency of plant extracts should be evaluated taking into account the purpose of the extract’s usage. Therefore, extracts for topical use, without a pronounced cytotoxic action, are considered as strong antibacterial agents, even if the MIC values are greater than 1 mg/mL [24]. Some authors declared strong activity of some herbal extracts in concentrations greater than 5 mg/mL [25]. Aiming to make our results applicable in the food industry, we have taken into account the fact that excess concentrations of extracts can affect the organoleptic food properties, and therefore, we did not use concentrations higher than 2.56 mg/mL in our study, but also included the lowest concentrations that would be comparable to antibiotics (1.25–40 µg/mL). Both celery extracts (CE1 and CE2) exhibited very uniform activity against almost all tested strains, particularly against *Listeria*, and the obtained MIC values were in accordance with previous research [16]. The obtained results were to be expected, taking into account the similarity of the chemical compositions of the analyzed celery extracts, although the limonene content was found to be slightly higher in CE1. The effect of CE1 on *Bacillus cereus* was rated as significant (MIC 160 µg/mL); data on the antibacterial activity of the supercritical extract of celery against *B. cereus* have not previously been published. With respect to the investigated parsley extracts, only PE1 showed a significant effect against *Bacillus* species (MIC 160–640 µg/mL), while against all other bacteria, PE1 showed weaker activity with a MIC of 2560 µg/mL. CE1, CE2 and PE1 showed anti-*Salmonella* activity with a MIC of 2560 µg/mL; to our knowledge, this is the first report on celery and parsley SFE activity against *Salmonella*. The stronger antibacterial activity of PE1 in comparison to PE2 was probably due to the presence of sedanolide, a substance known for its antibacterial activity [11,13,15,16,17] in the lipophilic fraction of PE1, as well as to the coextraction of fatty oils from the seed part of fruit that occurs at higher pressures.

Since the PE2 extract showed no antibacterial activity, and CE1 had slightly stronger activity than CE2, extracts CE1 and PE1 were selected for our cytotoxicity investigation. The cytotoxic effect was evaluated according to the findings of numerous authors, i.e., that a safe, noncytotoxic dosage was one whereby more than 50% cells stayed viable [26]. In our investigation, accordingly, even the highest investigated concentration of the CE1 and PE1 extracts, i.e., 2560 µg/mL, was not cytotoxic, due to survival rate remaining >50%. The concentration of 640 µg/mL was taken as the highest completely safe, applicable concentration for both of the extracts, with a survival rate of over 88% among the exposed cells. The cytotoxicity exerted at 640 µg/mL was considered negligible, given that in in vivo conditions, cells have repair mechanisms which are readily able to repair this amount of damage [26].

## 4. Materials and Methods

### 4.1. Materials

Seeds of celery (*Apium graveolens* L., cultivar Praski) and parsley (Petroselinum hortense Hoffm., cultivar Berlinski, medium long) were obtained in October, 2016, from the Institute of Field and Vegetable Crops, Novi Sad, Serbia. The moisture content of the plant material was 9.8% and 10% for celery and parsley, respectively. Commercial carbon dioxide (99% purity, Messer-Tehnogas, Belgrade, Serbia) was used for the SFE.

### 4.2. Preparation and Analytical Methods

#### 4.2.1. Supercritical Extraction

Extractions with SC CO_2_ were carried out in an Autoclave Engineers Screening System previously described in detail [27]. The Supercritical Extraction Screening System is designed for small batch research runs using CO_2_ as the supercritical medium with a maximum allowable working pressure of 41.3 MPa at 511 K (237.85 °C). Liquid CO_2_ is supplied from a CO_2_ cylinder by a siphon tube. The liquid CO_2_ is cooled in a cryostat between the cylinder outlet and the pump to prevent vaporization. The CO_2_ is pumped into the system until the required pressure is obtained. Backpressure regulators are used to set the system pressure (in the extractor and the separator). The extractor vessel (150 mL) is filled with the plant material (a fraction with an average particle diameter of 0.4 mm) from which a substance is to be extracted. Heating is applied to the extractor vessel for temperature elevation. The SC CO_2_ flows through the extractor and enters the separator vessel (500 mL). Samples of the extracted substance can be taken by opening the ball valve located at the bottom of the vessel. Extractions were performed at pressures of 10 and 30 MPa, and a temperature of 313 K (39.85 °C) until sample exhaustion. The flow rate of SC CO_2_ was 0.3 kg/h. Experiments were performed in triplicate.

#### 4.2.2. Gas Chromatography (GC)

Gas chromatography analysis of the extracts was carried out on a HP-5890 Series II GC apparatus (Hewlett-Packard, Waldbronn, Germany), equipped with split-splitless injector and automatic liquid sampler, attached to HP-5 column (25 m × 0.32 mm, 0.52 μm film thickness) and fitted to flame ionization detector (FID). The carrier gas flow rate (H_2_) was 1 mL/min, the split ratio was 1:30, the injector temperature was 250 °C, and the detector temperature was 300 °C, while the column temperature was linearly programmed from 40 °C to 260 °C (at rate of 4 °C/min) and then maintained isothermally at 260 °C for 10 min. Solutions of samples in chloroform were consecutively injected in amounts of 1 μL. Area percent reports, obtained by the standard processing of chromatograms, were used as the bases for quantification analyses. The response factor was considered to be 1.

#### 4.2.3. Gas Chromatography/Mass Spectrometry (GC/MS)

The same analytical conditions as those mentioned for GC were employed for GC/MS analysis, along with column HP-5MS (30 m × 0.25 mm, 0.25 μm film thickness), using HP G 1800C Series II GCD system (Hewlett-Packard, Palo Alto, CA, USA). Helium was used as carrier gas. Transfer line was heated at 260 °C. Mass spectra were acquired in EI mode (70 eV) in a *m/z* range of 40–450. The amount of 0.2 μL of sample solution in chloroform was injected. The components of the oil were identified by comparison of their mass spectra to those from the Wiley 275 and NIST/NBS libraries using different search engines. Calibration was performed using linear n-paraffins mixture (C6-C40) as a standard. The experimental values for retention indices were determined by the use of calibrated Automated Mass Spectral Deconvolution and Identification System Software (AMDIS ver. 2.1), compared to those from the available literature [28] and used as an additional tool to confirm the MS findings.

### 4.3. Antibacterial Activity of Plant Extracts

#### 4.3.1. Investigated Strains

The study included nonrepetitive isolates of *Bacillus cereus*, *B. subtilis*, *B. circulans*, *Staphylococcus aureus*, *Listeria greyi*, *L. seeligeri*, *L. welshimeri*, and *Salmonella* (*S.* Enteritidis). ATCC strains were also included in the study.

The choice of microorganisms was based on achieving a combination of food spoilage causative agents and foodborne pathogens. *Bacillus* and *Salmonella* were isolated by conventional microbiological methods, *Listeria* and staphylococci were isolated using the appropriate ISO standards [29,30]. Identification was done using a BBL Crystal Gram Positive ID kit (Becton Dickinson, Franklin Lakes, NJ, USA), API Listeria (bioMerieux, Marcy l’Etoile, France), API 20E (bioMeriuex). Appropriate diagnostic specific sera (Statens Serum Institute, Copenhagen, Denmark) were used for the serotyping of *Salmonella*. Investigated *Bacillus* strains were isolated from animal feedstuff samples and the environmental swabs taken from the surfaces in animal feed production plants. *Salmonella* was isolated from a clinical specimen originating from diseased animals. The specimen underwent routine microbiological diagnostics at the Department for Microbiology, Faculty of Veterinary Medicine, Belgrade, Serbia. *Staphylococcus aureus* and *Listeria* were isolated from a cheese specimen and frozen fish samples, which also underwent routine microbiologic analyses at the Department of Microbiology, Center for Food Analyses, Belgrade, Serbia.

#### 4.3.2. Investigation of Antibacterial Activity of Extracts

For the determination of the MIC values of plant extracts, cation adjusted Mueller Hinton II broth was used (CAMHB, Becton Dickinson). Dimethyl sulfoxide (DMSO, Merck Gesellschaft GmbH, Vienna, Austria) was used as a solvent for the herbal extracts. The broth microdillution method for antimicrobial susceptibility testing was applied in accordance with the CLSI standards [31,32]; instead of antibiotics, SFE extracts were applied. Ceftriaxone (Sigma Aldrich, city, country) and tetracycline (Carl Roth, Karlsruhe, Germany) were included as controls. The investigated concentrations of plant extracts were 2560, 1280, 640, 320, 160, 80, 40, 20, 10, 5, 2.5 and 1.25 expressed in μg/mL. Sedanolide (3-butyl-3a,4,5,6-tetrahydro-1(3*H*)-isobenzofuranone) with a purity of ≥95%, and (+)-Limonene (1-methyl-4*R*-(1-methylethenyl)-cyclohexene) with a purity of ≥98% (Cayman chemicals, Ann Arbor, MI, USA) were dissolved in DMSO and investigated at the same concentrations as the SFE extracts. The final bacterial inoculum density of 5 × 10^5^ CFU/mL was achieved by adding 5 μL of 1–2 × 10^7^ CFU/mL suspension of the investigated strain in microplate wells with 100 μL of previously added CAMHB. Microplates were incubated for 18–24 h at 37 °C.

### 4.4. Investigation of the Cytotoxic Effect of Extracts

The cytotoxic activity of celery and parsley extracts was determined using a MTT assay. The MTT assay is based on the mitochondrial enzyme reduction of tetrazolium dye to detect and determine cell viability [33,34]. Briefly, a specific mitochondrial enzyme of viable cells succinate dehydrogenase primary dark yellow color of the MTT (3-(4,5-dimethylthiazol-2-yl)-2,5 dipheniltetrazolium-bromide) dissolved in PBS (phosphate buffered saline) reduces blue to purple colored formazan. The cell membrane of viable cells is impermeable to formazan crystals, so they accumulate in the cells. During the analysis, the created formazan crystals must be dissolved using dimethyl sulfoxide (DMSO). The intensity of colors, which were obtained by dissolving formazan crystals, are quantitatively determined spectrophotometrically using an ELISA reader at different wavelengths.

Cell culture: MTT assay was done by using the RK 13 (rabbit kidney) cell line from the collection of the Department of Microbiology, Faculty of Veterinary Medicine, Belgrade. Cells were maintained in MEM with Earle’s Salts with l-glutamine (PAA, AUS) supplemented with 10% fetal bovine serum (PAA, AUS). Cells were incubated at 37 °C under 5% CO_2_/95% air in humidified atmosphere.

MTT reagent: MTT reagent (Invitrogen, Carlsbad, CA, USA) was dissolved at a concentration of 5 mg/mL in phosphate saline buffer-PBS (Invitrogen, USA) just before inoculation of the reagents in each well of the microtiter plates with the RK 13 cell lines and herbal oil.

MTT assay: RK cells were plated into 96-well plates. Incubation was performed at 37 °C under 5% CO_2_/95% air in a humidified atmosphere. After 24 h of incubation of the RK 13 cell line, a MTT assay was done. Briefly, 200 μL of different concentrations of plant extracts from 2560 mg/mL to 80 mg/mL dissolved in DMSO was added to each well of the microtitre plate. After 48 h exposure of RK 13 plant extract, 20 μL of MTT (5 mg/mL, Invitrogen, USA) was added to each well. Cells were incubated at 37 °C for 2 h; then, the medium was removed and 200 μL of DMSO was added to each well in order to dissolve the formazan crystals. After that, cells were incubated at 37 °C for 10 min, and then absorbance was read on LKB 5060-006 ELISA reader at a wavelength of 540 nm. Proper controls with a sterile medium and DMSO without the extracts were also established. The values of the blank wells were subtracted from each well of treated and control cells, and the percentage viability was calculated according to the following formula:

% viable cells = (the absorbance of the treated cells) − (the absorbance of the blank)/(the absorbance of the control) − (the absorbance of the blank) × 100(1)

LC50 values are the concentration of plant extract resulting in a 50% reduction of absorbance compared to untreated cells.

## 5. Conclusions

The present study has demonstrated the feasibility of using extraction with supercritical carbon dioxide at a temperature of 40 °C and pressures of 10 and 30 MPa to obtain extracts from celery fruits with significant antibacterial activity against *Bacillus*, *Listeria*, and *Staphylococcus aureus* strains. The parsley fruit supercritical extract obtained at 30 MPa showed weaker antibacterial activity against *Bacillus* spp. in comparison to celery extracts, and no antibacterial activity at the investigated concentrations against the remaining strains. The chemical compositions of the SC CO_2_ extracts were presented. The obtained results revealed that sedanolide was dominant in celery and apiol in parsley SC CO_2_ extracts. Pure sedanolide and limonene showed weaker antibacterial activity compared to supercritical celery extracts. The investigation of the cytotoxic effect of celery and parsley SF extracts obtained at 10 MPa indicated a maximum safe concentration of 640 µg/mL, suggesting their potential for safe application in the food industry.

## Figures and Tables

**Table 1:** Yields obtained in the SFE from celery and parsley fruits.

Plant	SFE Conditions	Yield (%)
Celery fruit	10 MPa/40 °C	2.70 ± 0.10
	30 MPa/40 °C	3.00 ± 0.15
Parsley fruit	10 MPa/40 °C	4.20 ± 0.08
	30 MPa/40 °C	9.30 ± 0.14

**Table 2:** Results of GC analysis of celery isolates.

Component	RI *	% (10 MPa) ***	% (30 MPa) ***
Limonene	1024	7.38	4.14
Pentyl Benzene	1152	0.65	0.43
Pinocarvyl acetate	1298	0.39	0.42
(*E*)-Caryophyllene	1417	0.26	0.25
β-Selinene	1489	6.12	6.10
α-Selinene	1498	1.01	1.01
Kessane	1529	0.39	-
Caryophyllene oxide	1582	0.34	-
3-Butyl phthalide	1647	9.13	9.31
Sedanenolide	1719	0.34	0.73
Sedanolide	1722	72.24	73.69
*n*-Hexadecanoic acid	1959	0.38	0.60
Bergaptene	2056	0.23	0.33
Oleic acid	2141	0.55	0.80

**Table 3:** Results of GC analysis of parsley isolates.

Component	RI *	% (10 MPa) ***	% (30 MPa) ***
Camphene	946	-	0.34
β-Pinene	974	-	0.56
Myrtenal	1195	0.37	0.44
Myristicin	1517	3.72	3.58
Elemicin	1555	0.29	-
2,3,4,5-Tetrametoxyallyl benzene	1568 **	0.95	-
Carotol	1594	0.37	-
Apiole	1677	86.08	88.90
Sedanenolide	1719	2.06	-
Murolan-3,9(11)-diene-10-peroxy	1729	0.36	-
Ledene oxide	2062	1.39	1.20
Graveolone	2155	0.86	0.88
Nonacosane	2900	2.09	3.35

* RI = retention index, experimentally determined by the use of calibrated Automated Mass Spectral Deconvolution and Identification System Software (AMDIS ver. 2.1) (Table 2 and Table 3): The calibration was performed using linear *n*-paraffins mixture (C_6_–C_40_) as standard. ** [19]. *** All the experiments were run in triplicate. The values represented an arithmetic mean (Table 2 and Table 3).

**Table 4:** Values of the minimal inhibitory concentrations of celery and parsley seed oils obtained by supercritical extraction.

Origin	Strain	CE1 ^a^ MIC (µg/mL)	CE2 ^b^ MIC (µg/mL)	PE1 ^c^ MIC (µg/mL)	PE2 ^d^ MIC (µg/mL)	Sedanolide MIC (µg/mL)	Limonene MIC (µg/mL)	Ceftriaxone MIC (µg/mL)	Tetracycline MIC (µg/mL)
ATCC 11778	*Bacillus cereus*	320	320	640	2560	1280	2560	8	8
Environment	*Bacillus cereus*	160	160	320	2560	1280	2560	8	16
Corn flour	*Bacillus cereus*	160	160	320	2560	1280	2560	8	8
Environment	*Bacillus subtilis*	640	320	n.d.	n.d.	1280	2560	n.d.	8
Environment	*Bacillus circulans*	160	80	160	320	1280	2560	16	2
MRSA ATCC 33591	*Staphylococcus aureus*	320	640	2560	>2560	2560	>2560	>256	4
ATCC ATCC 29213	*Staphylococcus aureus*	320	640	2560	>2560	2560	>2560	2	2
Cheese specimen 1	*Staphylococcus aureus*	160	320	2560	>2560	2560	>2560	2	4
Cheese specimen 2	*Staphylococcus aureus*	160	320	2560	>2560	2560	>2560	4	4
Cheese specimen 3	*Staphylococcus aureus*	320	640	2560	>2560	2560	>2560	2	8
Cheese specimen 4	*Staphylococcus aureus*	320	640	2560	>2560	2560	>2560	2	4
Cheese specimen 5	*Staphylococcus aureus*	320	640	2560	>2560	2560	>2560	4	8
Frozen fish specimen 1	*Listeria greyi*	640	640	2560	>2560	>2560	>2560	64	0,25
Frozen fish specimen 2	*Listeria seeligeri*	640	640	2560	>2560	>2560	>2560	64	0,5
Frozen fish specimen 3	*Listeria welshimeri*	320	640	2560	>2560	>2560	>2560	32	0,5
ATCC 19115	*Listeria monocytogenes*	640	640	2560	>2560	>2560	>2560	64	0,5
ATCC 13076	*Salmonella* Enteritidis	2560	2560	2560	>2560	>2560	>2560	n.d.	0,5
Diseased chicken 1	*Salmonella* Enteritidis	2560	2560	2560	>2560	>2560	>2560	n.d.	>512
Dead pigeon	*Salmonella* Enteritidis	2560	2560	2560	>2560	>2560	>2560	n.d.	>512
Diseased calf	*Salmonella* Enteritidis	2560	2560	2560	>2560	>2560	>2560	n.d.	>512

^a^—celery extract obtained at 10 MPa, ^b^—celery extract obtained at 30 MPa, ^c^—parsley extract obtained at 10 MPa, ^d^—parsley extract obtained at 30 MPa, n.d.—not determined.

**Table 5:** Cytotoxicity of the celery and parsley SFE extracts.

**Concentration of CE1 ^a^ Extract, µg/mL**	**% Viable Cells**	**% Dead Cells**
2560	57.95	42.04
640	89.03	10.96
320	100.00	0
**Concentration of PE1 ^b^ Extract, µg/mL**	**% Viable Cells**	**% Dead Cells**
2560	52.80	47.16
640	88.00	11.15
320	100.00	0

^a^—celery extract obtained at 10 MPa, ^b^—parsley extract obtained at 10 MPa.
